# Immunomodulatory effects of total intravenous and balanced inhalation anesthesia in patients with bladder cancer undergoing elective radical Cystectomy: preliminary results

**DOI:** 10.1186/1756-9966-32-6

**Published:** 2013-02-03

**Authors:** Maria Sofra, Paola Cordiali Fei, Luana Fabrizi, Maria Elena Marcelli, Claudia Claroni, Michele Gallucci, Fabrizio Ensoli, Ester Forastiere

**Affiliations:** 1 Department of Anesthesiology, Regina Elena, Rome National Cancer Institute, Via Elio Chianesi 53, 00144, Rome, Italy; 2 Department of Clinical Pathology and Microbiology, San Gallicano Dermatology Institute, Rome, Italy; 3 Department of Urology, Regina Elena, Rome National Cancer Institute, Rome, Italy

**Keywords:** Total intravenous anesthesia with target-controlled infusion (TIVA-TCI), Balanced inhalation anesthesia (BAL), Urinary bladder carcinoma, Cytokines, Tregs

## Abstract

**Background:**

Although surgery and anesthesia induce immunesuppression, remains largely unknown whether various anesthetic techniques have different immunosuppressive effects on cancer patients. Therefore, the aim of this study was to investigate the influence of total intravenous anesthesia with target-controlled infusion (TIVA-TCI) and balanced inhalation anesthesia (BAL) on the peri-operative levels of inflammatory cytokines and regulatory T cells (Tregs) in patients with bladder cancer undergoing surgery.

**Methods:**

Twenty eight consecutive patients with bladder cancer who underwent radical cystectomy were prospectively randomized into two groups to receive TIVA-TCI (n = 14) or BAL (n = 14). Before the induction of anesthesia (T0), 6–8 hours (T1) post-surgery, and 5 days post-surgery (T2), Tregs and serum levels of interleukin -1beta (IL-1β), interferon-gamma (IFN-γ), tumor necrosis factor-alpha (TNF-α), interleukin −2 (IL-2), interleukin −6 (IL-6), and interleukin −10 (IL-10) were measured.

**Results:**

In the peri-operative period all cancer patients showed a marked and significant increase in IL-6. Moreover, TIVA-TCI patients also showed a higher increase in IFN-γ, whereas in BAL patients Tregs were reduced by approximately 30% during surgery. The incidence of infections, metastases, and death was similar in both groups.

**Conclusions:**

The increase in the Th1 response in the TIVA-TCI group and the reduction in Tregs in the BAL group seem to balance the immunosuppressive effect induced by IL-6. Therefore TIVA-TCI and BAL can be both used in major surgery in patients with bladder cancer without worsening the outcome.

## Background

Increasing evidence supports the role of inflammation in tumor development, invasion, and metastasis, influencing the host immune response to the tumor [1]. This has been demonstrated in some tumors, particularly in bladder carcinoma, which is promoted by chronic inflammation and is uniquely sensitive to acute inflammation [2,3]. In addition, the surgical stress associated with general anesthesia causes immune suppression that accelerates the growth of neoplastic cells and premature enhanced metastasis [4-6].

Tumor-associated macrophages and T cells modify the microenvironment and are relevant to cancer progression. Tumor cell proliferation and invasion are also correlated with the release of specific cytokines [1,7]. Proinflammatory cytokines such as interleukin-6 (IL-6), tumor necrosis factor-alpha (TNF-α), and interleukin -1beta (IL-1β), which are released from tumor-infiltrating leukocytes, can activate signal transducers and activators of transcription protein 3 (STAT3), which induces immunosuppression that favors tumor cell proliferation [8,9]. T cells can exert both tumor suppression and cancer-promoting effects. Two subpopulations of lymphocytes have been described: those with Th1 or Th2 activity [10]. Th1 cells secrete pro-inflammatory cytokines, namely interferon-gamma (IFN-γ), and favor activation of macrophages and the inflammatory response. Th2 cells, with their pattern of cytokines interleukin-4 (IL-4) and interleukin-10 (IL-10), mediate the production of antibodies and have anti-inflammatory effects. In many tumors, such as colorectal cancer, melanoma, and pancreatic cancer, the Th1 response correlates with better prognosis [1,11,12]. Th1 cells probably exert a tumor suppressive effect also in bladder cancer [13]. Furthermore, induction of the T-helper type 1 immune response is required for effective bacillus Calmette-Guérin immunotherapy for bladder cancer [14].

Recent studies suggest that regulatory T cells (Tregs), a subpopulation of CD4+ T cells, play a fundamental role in maintaining immune tolerance [15-17]. Increasing evidence suggests that infiltrating and circulating Tregs inhibit antitumor immunity and promote tumor growth and disease progression, as observed in some clinical studies [18,19].

Nevertheless, only a few studies have evaluated the immunosuppressive effect of different anesthetic techniques in cancer patients undergoing major surgery. No guidelines for anesthesia procedures for cancer patients are available even though guidelines for operative procedures have been formulated for different types of cancer [20]. Previous studies on the role of inhaled and intravenous anesthetics in immune suppression showed contradictory results and appeared to be correlated with the type of cancer and surgery [20-23]. To our knowledge, no study has evaluated the effect of different anesthetic techniques in patients undergoing surgery for bladder cancer. Only Wang et al. [24] have assessed an increase in serum levels of IL-6 in patients undergoing radical cystectomy with intravenous anesthesia.

Thus, the aim of this study was to investigate the immunomodulatory effects of two established anesthetic techniques, total intravenous anesthesia with target-controlled infusion (TIVA-TCI) and balanced inhalation anesthesia (BAL), in patients with bladder cancer undergoing elective radical cystectomy and urinary bladder reconstruction via a Paduan ileal bladder, by studying changes in pro- and anti-inflammatory cytokines and Tregs.

## Methods

### Patient population

This study was approved by the Ethics Committee of the National Cancer Institute Regina Elena, Rome (Prot.CE/94/12), and written informed patient consent was obtained from all participants.

Between February 2010 and March 2011, 28 consecutive Caucasian patients with primary urothelial bladder cancer undergoing elective radical cystectomy were enrolled.

Patients with bladder cancer (22 males and 6 females, mean age 62.04 ± 8.63 years) were randomly assigned to receive either TIVA-TCI (n = 14) or BAL (n = 14). Randomization was based on a global assessment of anesthetic risk (ASA 1–2 vs. 3). A random code determined the anesthetic protocol. The surgeons, research assistants, medical staff, and nursing staff were blinded to the group assignment.

### Exclusion criteria

Exclusion criteria included: ASA >3, metabolic equivalent task <4, obesity, hemoglobin concentration <10 g/dl, endocrinologic, immunologic, and chronic infective diseases, diabetes, cortisone and immunosuppressive therapy, beta-blockers or angiotensin-converting enzyme inhibitor therapy, alcohol abuse, chronic liver disease, and chronic pain. None of the patients had received previous neo-adjuvant treatments (chemo, hormone, and radiotherapy).

### Anesthetic protocol

Thirty minutes before induction of anesthesia, all patients received 10 mg intramuscular ketoralac trometamina (Toradol™, Recordati, Milano, Italy) or 100 mg tramadolo cloridrato (Contramal™, AIC Formenti, Milano, Italy), 100 mg ranitidine (Ranidil™, Menarini, Firenze, Italy), and 0.5 mg atropine (Industria Farmaceutica Galenica Senese, Siena, Italy). Prior to starting anesthesia, a FloTrac pressure transducer was connected (Edwards Lifesciences, Irvine, CA) to the Vigileo system (Edwards Lifesciences, v.1.07) and inserted into a radial artery to monitor dynamic variables. In addition, central venous pressure and central venous oxygen saturation (ScvO_2_) were monitored from the right internal jugular vein.

Before starting surgery, patients in the TIVA-TCI group received a combination of propofol (Diprivan™, ASTRA-Zeneca, Milano, Italy) and remifentanyl (Ultiva™, GlaxoSmith-Kline AB, Verona, Italy). Propofol was administered with TCI though infusion pumps (Alaris PK CardinalHealth, Rolle, Switzerland). At induction, the target plasma dose was 4 mg/m and was decreased to 3 μg/ml during the operation. Remifentanil was administered as a continuous intravenous infusion. At induction, the dose was 0.25 μg kg^-1^ min^-1^, and it was lowered to 0.15 μg kg^-1^ min^-1^ during surgery. This combination was modified by 0.05-μg kg^-1^ min^-1^ steps according to analgesic needs and hemodynamic parameters.

In the BAL group, patients received inhalation anesthesia with sevoflurane/O_2_/air (Sevorane™, Abbott, Latina, Italy) throughout the entire surgery. Before induction of anesthesia, 1–2 μg/kg fentanyl (Fentanest™, Pftzer, Latina, Italy) was administered. Anesthesia was induced by 0.1-0.2 mg/kg midazolam (Hameln pharmaceuticals Gmbh, Hameln, Germany), and the inhalation anesthesia was comprised of a mixture of sevoflurane/O_2_/air. For maintenance, the end-tidal sevoflurane concentration was kept at 1.4-2.8 vol %.

In both groups, 0.1-0.5 mg/kg cisatracurium besylate (Nimbex™, Glaxo Smith Kline) was given to facilitate orotracheal intubation, followed by the continuous application of 0.06-0.12 mg kg^-1^ h^-1^ cisatracurium via infusion pumps. The lungs were mechanically ventilated in a volume-control mode with settings aimed at achieving normocapnia, reaching a tidal volume up to 8–10 ml/kg and a respiratory frequency of 10–12 breaths/min. Mechanical ventilation was initiated with a mixture of 50% O_2_ and 50% air, and the inspired oxygen concentration was 40% during surgery. All patients were kept supine during the operation. No patient received inotropes, vasopressors or methoclopramide during or after surgery.

Monitoring included evaluation of cardiac hemodynamic parameters (electrocardiogram, heart rate, invasive blood pressure, systolic, diastolic, mean blood pressure [MAP], central venous pressure, stroke volume variation, cardiac index); tissue perfusion markers (ScvO_2_, O_2_ delivery index, arterial lactates, base excess, diuresis), respiratory parameters (pulse oximetry, end-tidal CO_2_, airway pressure, end-tidal sevoflurane), esophageal temperature, and blood glucose. The type of fluids (colloid and crystalloid) and the total volume were administered according to the goals optimized for a Cardiac Index >2.5 L/min/m^2^, MAP >90-105 mmHg, and Oxygen Delivery Index >600 ml/min/m^2^. Furthermore, the ScvO_2_ value was maintained at ≥70%. Patients received 1 packed red cell unit for each 1 g/dl of hemoglobin when its value was <8 g/dl.

After surgery, the residual neuromuscular blockade was reversed with a mixture of atropine and neostigmine (Intrastigmina™, Lusofarmaco, Milano, Italy) only if deemed clinically necessary. Anesthetic agents were switched off, and 100% O_2_ was given with 8 l/min fresh gas flow for 1 min. Supplemental oxygen was not given postoperatively.

Hypothermic prevention during anesthesia was achieved by warm venous infusion (warmed serum), and a thermal blanket was applied to cover the upper part of the body. In addition, a warming forced-air blanket was used post-surgery (Equator Covective Warming™, Smith Medical Italia, Milano, Italy).

After tracheal extubation, all patients received an intravenous bolus of 2 mg morphine (Recordati). A patient-controlled analgesia device (Deltec™ , Smiths Medical ASD, St Paul, MN) was then connected to an intravenous infusion device and was set to deliver 1 mg morphine with a 7-min lockout time. Patient-controlled analgesia was maintained until daily morphine consumption was <10 mg. In addition, patients received 20 mg ketoralac for 3 days or 100 mg tramadolo cloridrate for 1 day.

### Peri-operative protocol

Before the induction of anesthesia (T0), 6–8 hours post-surgery (T1), and 5 days post-surgery (T2), blood samples were drawn to determine immunologic parameters, including Tregs and the serum concentration of IL-1β, IFN-γ, TNF-α, IL-2, IL-6, and IL-10.

The following clinical parameters were evaluated: (a) histological type and pathological tumor-node-metastasis stage, (b) quantity and type of liquids administered, (c) blood loss, (d) transfusion of allogenic blood and/or autotransfusion, (e) pre and post-operative complications such as hypertension, hyperglycemia, hypothermia, and pain (evaluated by a 6-point verbal rating scale: 0: no pain to 5: most severe pain imaginable), (g) post-operative infection rate.

Furthermore, follow-up was performed to assess the disease-free interval, metastasis, and survival of each patient.

### Serological parameters

The serum levels of different cytokines were measured with enzyme immunoassays (IL-2 and IL-10, Boster Biological Technology, CA, USA) or multiparametric assays based on chemiluminescent detection of a cytokine array. The latter allows simultaneous detection of multiple molecules (IL-6, IFN-γ, TNF-α, IL-1β; Human cytokine array and SignaturePLUS™ CCD Imaging & Analysis System, Aushon Biosystem, MA, USA).

### Evaluation of tregs

Peripheral blood mononuclear cells were isolated by gradient centrifugation, and Tregs were identified by the expression of CD4 and CD25 on the cell membrane and by FoxP3 intracellular staining using flow cytometry as previously described [25]. (Both the detecting antibodies and the FacsCalibur flow cytometer were from BD Biosciences, San Jose, CA).

### Statistical analysis

Data were analyzed with Statistical Package for the Social Sciences (SPSS) 14.0 software. Continuous and categorical variables were expressed as the mean ± standard deviation or standard error and as frequency values and proportions, respectively. Pearson’s chi-square test was used to assess possible differences in dichotomous variables between the various groups examined. The means of normally distributed data were compared with the Student’s *t-*test. In the other cases, the groups were compared with the Mann-Whitney’s *U* test. P values of the tests were adjusted using the Bonferroni method. Paired samples were analyzed by *t*-test and Wilcoxon Signed Ranks Test. A p-value of <0.05 was considered statistically significant.

## Results

### Clinical characteristics of the patients

The clinical characteristics of the patients enrolled in the study are reported in Table 1. No significant differences were observed regarding age or gender between TIVA-TCI and BAL cancer patients. Eighteen out of 28 patients who underwent surgery (64.3%) had an ASA I-II (9 in each group), whereas 10 (35.7%) had an ASA III. The mean duration of anesthesia was 3.27 ± 0.48 h, with no differences between the TIVA-TCI and BAL groups (p = 0.42). All patients showed a high grade urothelial carcinoma (G3). No significant differences between the two groups were observed regarding tumor size, invasiveness (pT), lymph node involvement (pN), body mass index, time of surgery and hospitalization. 

**Table 1:** Clinical characteristics of patients with bladder cancer who underwent radical cystectomy with TIVA-TCI or BAL anesthesia

	**All cancer patients (n. 28)**	**TIVA-TCI (n. 14)**	**BAL (n. 14)**	**P TIVA-TCI vs. BAL**
**Age (yrs)**	62.04 ± 8.63	63.2 ± 6.8	61.2 ± 10.8	0.57
**Sex , n (%)**				
males	23 (82.1%)	12 (85.7%)	11 (78.6%)	0.62
females	5 (1*7*.9%)	2 (14.3%)	3 (21.4%)	
**Histological type of cancer**				
High grade urothelial carcinoma	28 (100%)	14 (100%)	14 (100%)	1.00
**pT, n (%)**				
1-2	11 (39.3%)	6 (42.9%)	5 (35.7%)	0.70
3	17 (60.7%)	8 (57.1%)	9 (64.3%)	
**pN, n (%)**				
0	22 (78.6%)	12 (85.7%)	10 (71.4%)	0.34
1	2 (7.1%)	0	2 (14.3%)	
2	4 (14.3%)	2 (14.3%)	2 (14.3%)	
**ASA, n (%):**				
I-II	18 (64.3%)	9 (64.3%)	9 (64.3%)	1.00
III	10 (35.7%)	5 (35.7%)	5 (35.7%)	
**Weight (BMI )**	25.8 ± 4.2	27.1 ± 5.9	25.1 ± 3.0	0.55
**Time of surgery (h)**	3.12 ± 0.59	3.08 ± 0.58	3.17 ± 0.56	0.27
**Time of anaesthesia (h)**	3.27 ± 0.48	3.18 ± 0.45	3.35 ± 0.51	0.42
**Time of hospitalization (days)**	13.29 ± 1.00	13.58 ± 0.99	13.00 ± 0.95	0.16
**Metastasis after surgery, n (%)**	4 (14.3%)	1 (7.1%)	3 (21.4%)	0.28
**Death from cancer, n (%)**	5 (17.9%)	1 (7.1%)	4 (28.6%)	0.14
**Death from any cause, n (%)**	7 (25.0%)	2 (14.3%)	5 (35.7%)	0.19

Values are expressed in absolute values or mean ± SD.

During surgery, decreases in hematocrit and hemoglobin concentration were observed in both groups, but intra-operative blood loss was similar (Table 2). Transfusion of allogenic blood and autotransfusion were performed in 11 and 6 patients, respectively (5 and 3 in the TIVA-TCI group and 6 and 3 in the BAL group, respectively), with no significant differences in the number of transfusions between groups. Also, the volume of electrolyte solution administered during anesthesia was similar in the TIVA-TCI and BAL groups (Table 2). Similarly, no statistical differences were observed between groups regarding hemodynamic and respiratory parameters, tissue perfusion markers, temperature, or glucose levels (Table 2). 

**Table 2:** Perioperative clinical data of patients with bladder cancer who underwent radical cystectomy with TIVA-TCI or BAL anesthesia

	**TIVA ( n. 14)**	**BAL (n. 14)**	**P TIVA vs. BAL**
**HB (g/dl)**			
Pre-anaesthesia	13.51 ±1.80	14.42 ± 1.33	0.14
Intraoperative	9.82 ±1.63	10.43 ± 1.82	0.47
5 days post-surgery	9.63 ±1.24	9.70 ± 1.35	0.86
**HCT (%)**			
Pre-anaesthesia	39.53 ± 5.23	42.55 ± 4.47	0.14
Intraoperative	28.2 ±5.12	30.33 ± 5.41	0.52
5 days post-surgery	29.16 ±4.85	28.32 ± 3.80	0.65
**Blood loss (ml)**	1596 ± 365	1539 ± 418	0.70
**Total amount of crystalloid received (ml)**	3250 ± 1513	2875 ± 772	0.49
**Total amount of colloid received (ml)**	350 ± 250	300 ± 250	0.61
**Blood transfusion (n)**	1.15 ± 1.64	1.22 ± 1.71	0.96
**Intraoperative autotransfusion (n)**	0.47 ± 0.71	0.33 ± 0.62	0.82
**Intraoperative body temperature (°C)**	36.14 ± 0.22	36.24 ± 0.26	0.93
**Intraoperative blood glucose (mg/dl)**	120.04 ± 21.38	116.63 ± 23.61	0.72
**Intraoperative MAP (mmHg)**	103.66 ± 12.82	106.41 ± 12.13	0.60
**Intraoperative CVP(cm H**_**2**_**O)**	10.32 ± 1.23	10.14 ± 1.33	0.75
**Intraoperative SpO**_**2**_**(%)**	97.60 ± 0.92	96.61 ± 2.82	0.30
**Arterial lactate level (mmol/l)**			
1 h post-surgery	0.82 ± 0.22	0.61 ± 0.34	0.82
6 h post-surgery	1.77 ± 0.32	1.87 ± 0.25	0.83
5 days post-surgery	1.32 ± 0.35	1.27 ± 0.22	0.91
**Intraoperative BE (mmol/l)**	0.32 ± 0.51	0.43 ± 0.38	0.53
**Intraoperative PaO**_**2**_**(mmHg)**	222.21 ± 10.23	215.11 ± 23.11	0.73
**Pain (Verbal Rating Scale)**			
1 h post-surgery	1.32 ± 0.62	1.22 ± 0.81	0.59
6 h post-surgery	1.14 ± 0.44	1.07 ± 0.51	0.54
5 days post-surgery	0.73 ± 0.56	0.82 ± 0.64	0.46

Values are presented as mean ± SD.

None of the patients experienced adverse events during their postoperative course such as pulmonary infections requiring antibiotic treatment, systemic inflammatory response syndrome, sepsis, acute respiratory distress syndrome, or surgical revision.

Metastases after surgery were observed in only 4 out of 28 cancer patients (14.3%): one in the TIVA-TCI group and 3 in the BAL group (p = 0.28) (Table 1). No significant differences were observed in the incidence of death from any cause or tumors between the TIVA-TCI and BAL groups, even though the number of patients who had died was higher in the BAL group (4 in BAL vs. 1 in TIVA-TCI, p = 0.14) (Table 1).

### Changes in concentrations of inflammatory cytokines

**Figure 1:**
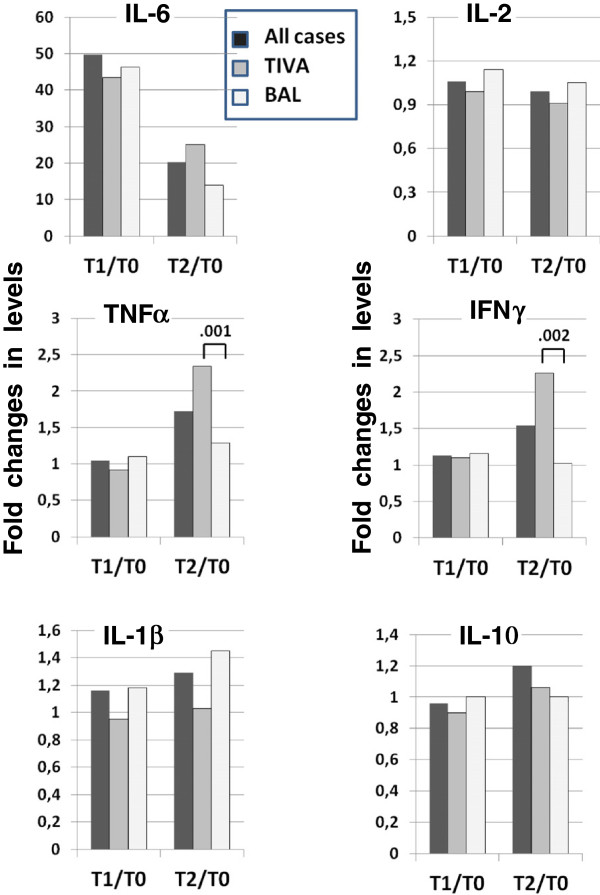
**Changes in cytokine levels between T0 (before the induction of anesthesia) and T1 (6–8 hours post-surgery) and between T0 and T2 (5 days post-surgery) in all cases, in TIVA-TCI, and BAL. **TIVA-TCI and BAL patients showed a marked and significant increase in IL-6 at T1 compared to values prior to surgery (T0) (p = 0.005), with an increase of about 50 times. These values were reduced at T2, but remained about 10 times higher than baseline values (p = 0.005). There were no significant differences between the TIVA-TCI and BAL groups. The TIVA-TCI group showed a significant increase in TNF-α levels between T2 and T0 compared to the BAL group (2.34 vs. 1.29 times, p = 0.001). At T1, differences were not statistically significant due to the high variability observed. Similarly, the increase in IFN-γ observed at T2 was significantly different in patients undergoing TIVA-TCI anesthesia compared to BAL. IFN-γ levels showed an increase of 2.26 times at T2 compared to T0 in the TIVA-TCI group and only 1.03 times in the BAL group (p = 0.002). The values of other cytokines remained constant during the three measurements in both groups.

TIVA-TCI patients showed a marked and significant increase in IL-6 at T1 (6–8 hours post-surgery), reaching a value of 132.6 ± 37.9 pg/ml compared to the value of 5.3 ± 4.4 pg/ml measured before surgery (T0; p = 0.005), an increase of about 50-fold (Table 3, Figure 1). These values were reduced 5 days post-surgery (T2), but remained about 10-fold higher than baseline values (p = 0.005). Even in the BAL group, we observed a similar increase at T1 (132.4 ± 53.9 pg/ml vs. 4.2 ± 3.3 pg/ml, p = 0.005) that was followed by a reduction at T2 that remained about 10 times higher than baseline values (p = 0.005) (Table 3). No significant differences were found between TIVA-TCI and BAL groups in the levels of IL-6 just before surgery or peri-operatively. 

**Table 3:** Changes of immunologic parameters before induction of anaesthesia (T0), 6–8 hours post-surgery (T1) and 5 days post-surgery (T2) in patients who underwent TIVA-TCI and BAL anesthesia

	**T0**		**T1**		**T2**	
	**TIVA-TCI**	**BAL**	**TIVA-TCI**	**BAL**	**TIVA-TCI**	**BAL**
**IL-1β (pg/ml)**	0.58 ± 0.53	0.59 ± 0.53	0.57 ± 0.48	0.62 ± 0.52	0.60 ± 0.53	0.69 ± 0.50
**IFN-γ (pg/ml)**	0.55 ± 0.48	0.57 ± 0.41	0.53 ± 0.42	0.58 ± 0.51	1.07 ± 0.48 (p)	0.58 ± 0.58 (p)
**TNF-α (pg/ml)**	0.94 ± 0.64	0.76 ± 0.74	0.72 ± 0.45	0.98 ± 1.01	1.88 ± 1.18 (q)	1.28 ± 1.10 (q)
**IL-2 (pg/ml)**	20.99 ± 4.22	21.33 ± 5.10	20.24 ± 3.02	23.38 ± 6.22	18.46 ± 2.30	21.21 ± 6.70
**IL-6 (pg/ml)**	5.35 ± 4.37 (a,b)	4.28 ± 3.27 (e,f)	132.59 ± 37.91 (a)	132.81 ± 54.23 (e)	53.60 ± 111.20 (b)	40.76 ± 50.82 (f)
**IL-10 (pg/ml)**	1.50 ± 0.21	1.48 ± 0.15	1.46 ± 0.31	1.50 ± 0.16	1.55 ± 0.29	1.51 ± 0.21
**Leucocytes (%)**	7.79 ± 3.22	9.30 ± 4.73	11.98 ± 3.99	13.09 ± 4.65	9.54 ± 2.25	9.14 ± 3.57
**Lymphocytes (%)**	16.76 ± 11.23 (c,d)	12.94 ± 12.33 (l)	6.02 ± 5.45 (c)	7.97 ± 6.36 (l,m)	8.45 ± 8.66 (d)	11.80 ± 9.19 (m)
**Tregs (%)**	3.01 ± 1.16	3.34 ± 1.75 (n,o)	2.69 ± 0.97	2.45 ± 2.22 (n)	2.79 ± 1.32	2.41 ± 1.27 (o)
**Neutrophils (%)**	48.30 ± 30.42	54.11 ± 22.27	67.56 ± 31.16	62.70 ± 30.54	58.50 ± 28.09	63.30 ± 20.23
**Monocytes (%)**	5.34 ± 4.40	5.64 ± 3.36	4.58 ± 3.67	4.57 ± 3.74	6.61 ± 4.14	6.65 ± 3.82
**Eosinophils (%)**	1.73 ± 1.26	4.98 ± 4.46 (g)	1.17 ± 3.05	0.80 ± 1.38 (g,h)	2.23 ± 1.63	4.65 ± 2.87 (h)
**Basophils (%)†**	1.30 ± 2.45	0.48 ± 0.27 (i)	0.22 ± 0.16	0.20 ± 0.27 (i)	0.60 ± 0.48	0.37 ± 0.24

Values are presented as mean ± SD.

Statistical analysis:

TIVA-TCI : (a) T0 vs T1 p = 0.005, (b)T0 vs T2 p = 0.005; (c) T0 vs T1 p = 0.01, (d) T0 vs T2 p = 0.05.

BAL : (e) T0 vs T1 p = 0.005, (f) T0 vs T2 p = 0.005; (g) T0 vs T1 p = 0.005, (h) T1 vs T2 p = 0.002; (i) T0 vs T1 p = 0.01; (l) T0 vs T1 p = 0.04, (m) T1 vs T2 p = 0.03 ; (n) T0 vs T1 p = 0-02, (o)T0 vs T2 p = 0.03.

TIVA-TCI vs. BAL : (p) p = 0.01 ; (q) p = 0.04.

The TIVA-TCI group showed a significant increase in TNF-α levels between T2 and T0 compared to the BAL group (2.34 vs. 1.29 times, respectively, p = 0.001). At T1, the levels of TNF-α were not significantly different, possibly due to the high variability observed. The values of IL-2 remained constant during the three measurements (Figure 1).

Th1 pro-inflammatory activities were evaluated by measuring plasma levels of IFN-γ. Although we observed only slight changes between T0 and T1, the differences became significant at T2 when there was an increase by 1.5-fold compared to T0. The increase in IFN-γ observed at T2 was significantly different in patients undergoing TIVA-TCI compared to BAL (Figure 1). In fact, IFN-γ levels showed a mean increase of 2.26-fold at T2 in the TIVA-TCI group and only 1.03-fold in the BAL group (p = 0.002).

There were no significant changes in Th2 activity just before surgery and peri-operatively, as assessed by IL-10 levels (Figure 1).

### Changes in circulating blood cells

Some changes in blood cells were observed during anesthesia and surgery. Both TIVA-TCI and BAL patients showed a significant reduction in lymphocytes at T1 (p = 0.01 and p = 0.04, respectively) that slightly increased at T2 (Table 3).

Interestingly, the BAL group showed a significant reduction in Tregs (p = 0.02) at T1, which was maintained at T2 (T0 vs. T2, p = 0.03) (Table 3). In contrast, TIVA-TCI patients showed no changes in Treg levels just before surgery and postoperatively (Table 3).

The reduction in circulating lymphocytes and Tregs at T1 was associated with a significant reduction in eosinophils (p = 0.005) and basophils (p = 0.01) in the BAL group, and these values returned to baseline values at T2 (Table 3). Because no other changes in leukocytes or monocytes were demonstrated, the reported modifications of lymphocytes we observed appear to be independent of the hemodilution.

## Discussion

The results of our study show that all patients with bladder cancer showed a notable increase in IL-6 peri-operatively. In patients undergoing TIVA-TCI anesthesia, the increase in IL-6 was also associated with a significant increase in the pro-inflammatory Th1 cytokine IFN-γ. In contrast, in BAL patients Tregs were reduced by about 30% during surgery and remained low up to 5 days after surgery (Table 3, Figure 1).

Our study suggests that the marked increase in serum IL-6 observed in the early post-operative period is not related to the type of anesthesia and pain, but appears to be mainly related to surgical stress as demonstrated by previous studies [22,26,27]. It has been hypothesized that release of IL-6 during surgical stress determines the release of catecholamine and glucocorticoids, which induce immune suppression [4,28]. The immunosuppressive effect was also observed in our cases by the reduction in circulating lymphocytes at T1, which persisted at T2 and was independent of the type of anesthetic used.

Previous studies regarding the immune suppressive effect of inhaled and intravenous anesthetics have been contradictory [20-23]. Our results are in agreement with findings of a recent study by Kvarnsrtom et al. [26], who observed a similar increase in IL-6 levels in the peri-operative period in cancer patients undergoing major abdominal surgery who were randomized to receive either propofol-remifentanil or sevoflurane-fentanyl. An increase in IL-6 was also observed in patients without cancer undergoing anesthesia with sevoflurane [29,30]. Conversely, others studies have reported low serum levels of IL-6 in patients given TIVA anesthesia in comparison to inhaled anesthetics [31] and in some experimental studies after administration of propofol [32]. Other studies reported a suppression of the production of IL-6 by sevoflurane [33,34].

It is currently unclear whether these differences are due to the type of cancer or surgery or depend on other still unrecognized causes. This is the first study to evaluate changes in the levels of cytokines induced by two different anesthetic techniques in patients with bladder cancer undergoing cystectomy. A few earlier studies had shown a greater increase in IL-6 in patients undergoing open radical cystectomy compared to those who underwent laparoscopy [24,35]. IL-6 is a multifunctional cytokine that supports cancer cell proliferation and inhibits apoptosis through activation of STAT3 [36,37]. The role of IL-6 in human cancer is reinforced by the observation of elevated serum levels of IL-6 and soluble IL-6 receptor in patients with bone metastasis and a poor clinical outcome [38]. Additionally, IL-6 acts as an angiogenic factor [38]. IL-6 and STAT are activated by modifications in MAP and O_2_ saturation [39]. The fact that in our study MAP and O_2_ saturation were constant in both techniques confirms the hypothesis that the increase in IL-6 is specifically related to surgical manipulation and anesthesia and is independent of the type of anesthetic drugs used. In the BAL group, at the end of surgery (T1) (Table 3), and in the TIVA-TCI group, after 5 days (T2), an increase in TNF-α levels was also observed (Table 3). It is important to note that increased TNF-α expression has been reported in recurrent, larger bladder tumors as well as in tumors that show progression in grade and stage [40].

The immunosuppressive effect of surgery produces a reduction in progression-free survival and an increase in developing infections in the peri-operative period [23]. Because no patients in our study showed infections in the postoperative week with either anesthetic technique, we hypothesize that there was an immunomodulatory effect that antagonized the immunosuppression and was likely correlated with the increase in IL-6 levels during surgery. In addition, non-statistically significant differences in progression-free survival, overall survival, and occurrence of metastases were observed between the TIVA-TCI and BAL groups. This immunomodulatory effect occurred with both anesthetic techniques albeit in different ways.

A significant increase in the Th1 response was found in patients undergoing TIVA-TCI anesthesia. This finding was illustrated by the fact that 5 days after surgery (T2), the levels of IFN-γ increased 2.26-fold compared to pre-surgery values (Figure 1). Because IL-10 levels remained constant in both groups, the Th2 response did not appear to be altered by either anesthetic procedure.

Th1 cells probably exert a tumor suppressive effect in bladder cancer [13]. In fact, in bladder tumor patients, a marked polarization exists towards the expression of Th2-type cytokines, whereas Th1 remains suppressed. Th1 cytokines play an important role in bacillus Calmette-Guérin (BCG)-induced macrophage cytotoxicity, and the combination of BCG with select Th1-stimulating cytokines may enhance the effect of BCG in the treatment of bladder cancer patients [41].

In patients undergoing BAL anesthesia, a significant reduction in Treg levels of 30% was observed in the early peri-operative period (T1) (p = 0.03; Table 3) and remained constant up to T2, showing values similar to those measured in healthy controls. This is the first study to evaluate the effect on circulating levels of Tregs due to various types of anesthesia. Earlier evidence suggested that Tregs accumulate in tumors and in the peripheral blood of patients with cancer and through suppression of the anti-tumor immune response these cells promote tumor growth and disease progression in a variety of human malignancies, including bladder cancer [18,19,42]. The role of Tregs in metastasis is just beginning to emerge, and circulating Tregs are associated with poor prognosis in some human cancers [43]. In vivo expansion of Tregs is mediated by glucocorticoid-induced tumor necrosis factor receptor family-related (GITR) proteins [44]*.* Interestingly, Tregs detected in tumor tissues express high levels of GITR molecules. Depletion of Tregs by anti-GITR mAb represents a novel mechanism for cancer immunotherapy [45]. Therefore, the reduction in Tregs we observed in the BAL group appears particularly remarkable in patients with bladder cancer, a type of neoplasm that is responsive to immunotherapy.

## Conclusions

The increase in the Th1 response observed in the TIVA-TCI group and the reduction in Tregs observed in BAL patients seem to balance the putative immunosuppressive effect induced by IL-6 and supports the hypothesis that TIVA-TCI and BAL techniques can be both used during major surgery in patients with bladder cancer without worsening the outcome.

## Competing interests

M Sofra, P Cordiali Fei, L Fabrizi, ME Marcelli, C Claroni, M Gallucci, F Ensoli and E Forastiere: *No interest declared*.

## Authors’ contributions

MS and EF have made contribution to conception and design of the study, acquisition, analysis and interpretation of data. PCF has made contribution to acquisition, analysis and interpretation of data. LF, MEM, CC, MG and FE have made contribution to acquisition of data, All Authors have been involved in drafting the manuscript or revising it critically for important intellectual content and have given final approval of the version to be published. All authors read and approved the final manuscript.

## Funding

This work was supported by a grant from “Istituto Nazionale Tumori Regina Elena” and “Ministero della Salute” for the Research project “Anesthesia and Immunity.”
